# 'Le Rouge et le Noir': A decline in flavone formation correlates with the rare color of black dahlia (*Dahlia variabilis* hort.) flowers

**DOI:** 10.1186/1471-2229-12-225

**Published:** 2012-11-23

**Authors:** Jana Thill, Silvija Miosic, Romel Ahmed, Karin Schlangen, Gerlinde Muster, Karl Stich, Heidi Halbwirth

**Affiliations:** 1 Technische Universität Wien, Institut für Verfahrenstechnik, Umwelttechnik und Technische Biowissenschaften, Getreidemarkt 9/1665, Wien, A-1060, Austria

**Keywords:** *Dahlia variabilis* hort, Asteraceae, Flower colour, Black flower, Flavonoids, Anthocyanins, Flavones, Flavone synthase II, Transcription factors

## Abstract

**Background:**

More than 20,000 cultivars of garden dahlia (*Dahlia variabilis* hort.) are available showing flower colour from white, yellow and orange to every imaginable hue of red and purple tones. Thereof, only a handful of cultivars are so-called black dahlias showing distinct black-red tints. Flower colour in dahlia is a result of the accumulation of red anthocyanins, yellow anthochlors (6’-deoxychalcones and 4-deoxyaurones) and colourless flavones and flavonols, which act as copigments. White and yellow coloration occurs only if the pathway leading to anthocyanins is incomplete. Not in all cultivars the same step of the anthocyanin pathway is affected, but the lack of dihydroflavonol 4-reductase activity is frequently observed and this seems to be based on the suppression of the transcription factor *DvIVS*. The hitherto unknown molecular background for black colour in dahlia is here presented.

**Results:**

Black cultivars accumulate high amounts of anthocyanins, but show drastically reduced flavone contents. High activities were observed for all enzymes from the anthocyanin pathway whereas FNS II activity could not be detected or only to a low extent in 13 of 14 cultivars. cDNA clones and genomic clones of FNS II were isolated. Independently from the colour type, heterologous expression of the cDNA clones resulted in functionally active enzymes. *FNS II* possesses one intron of varying length. Quantitative Real-time PCR showed that *FNS II* expression in black cultivars is low compared to other cultivars. No differences between black and red cultivars were observed in the expression of transcription factors *IVS* and possible regulatory genes *WDR1, WDR2, MYB1, MYB2, 3RMYB* and *DEL* or the structural genes of the flavonoid pathway. Despite the suppression of *FHT* expression, flavanone 3-hydroxylase (FHT, synonym F3H) enzyme activity was clearly present in the yellow and white cultivars.

**Conclusions:**

An increased accumulation of anthocyanins establishes the black flowering phenotypes. In the majority of black cultivars this is due to decreased flavone accumulation and thus a lack of competition for flavanones as the common precursors of flavone formation and the anthocyanin pathway. The low FNS II activity is reflected by decreased *FNS II* expression.

## Background

**Figure 1:**
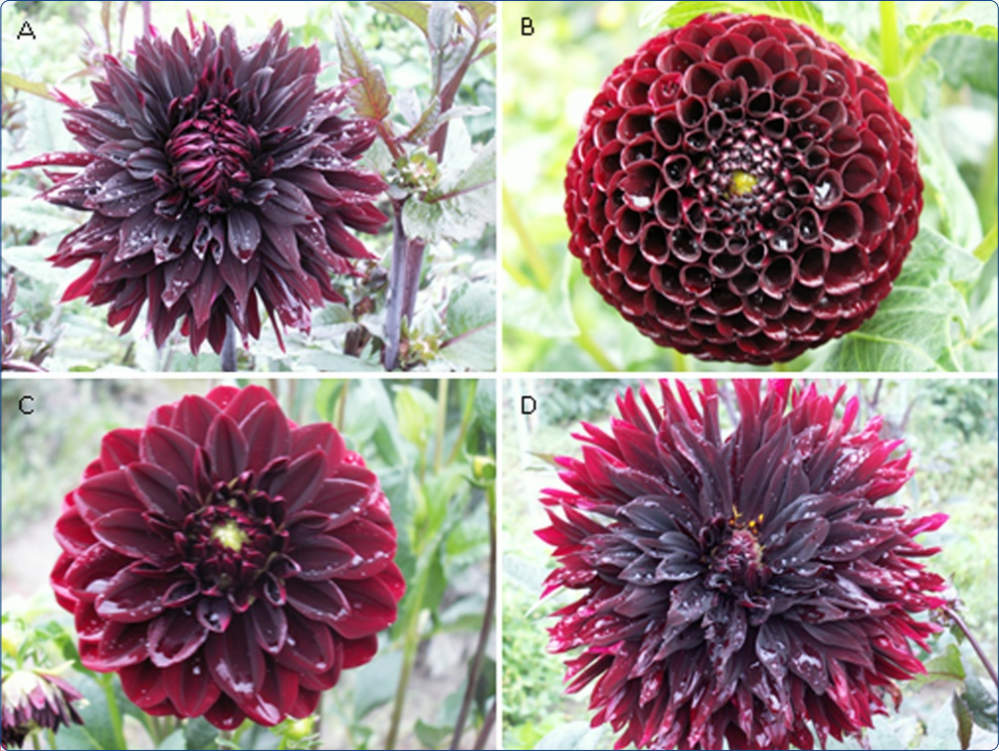
**Examples of black dahlia cultivars. A**: cv. Karma Choc, **B**: cv. Tisa, **C**: cv. Arabian Night, **D**: cv. Kenora Macop B. Photos of other cultivars can be found e.g. in the dahlia catalog at http://www.dahlie.net/ or http://www.dahlienwirth.at/dahliensorten.html.

Dahlia (*Dahlia variabilis* hort.), a member of the Asteracea family, is a popular garden flower. More than 20,000 cultivars are available world-wide [1]. Garden dahlias are hybrids, which are classified according to shape and size of the plant and its inflorescences. A broad spectrum of flower colours exists ranging from white and yellow to almost every imaginable hue of red, orange and magenta (red tones), but blue flower colour does not occur [1]. In contrast to the multitude of available red-shaded cultivars, only a handful of so-called black cultivars exist (Figure 1). Such cultivars frequently have expressive names e.g. Chat Noir (French term for black cat), Magic Night, Arabian Night and Black Jack.

Dahlia flower colour is exclusively based on the accumulation of flavonoids and biochemically related anthochlors (chalcones, aurones) [2-4]. The study of pigments in dahlia started in 1956, when the presence of anthocyanins, flavones, flavonols, chalcones and aurones were elaborately described in *Dahlia* species [2,5]. 6’-Deoxychalcones (derivatives of butein and isoliquiritigenin) and the corresponding 4-deoxyaurones (derivatives of sulfuretin) are the chemical base of yellow flower colour in *D. variabilis* and are mixed with anthocyanins (derivatives of pelargonidin and cyanidin) in orange and red forms [6,7]. A screening of more than 200 dahlia cultivars showed that the different red tones are based on the same set of anthocyanins and that variation in the anthocyanin concentration, the modification pattern of the core structures and probably also pH is responsible for the formation of different hues [3]. Orange, rose and lilac cultivars frequently showed lower anthocyanin contents than red and magenta cultivars. In the case of rose and lilac cultivars this seems to be primarily based on a lower chalcone synthase (CHS) activity [3]. Yellow and white cultivars do not accumulate anthocyanins due to a bottleneck or blockage of the anthocyanin pathway. Although there is no general rule, for yellow cultivars this was most frequently observed at the levels of both flavanone 3-hydroxylase (FHT, synonym F3H) and dihydroflavonol 4-reductase (DFR) [3], which was recently confirmed by gene expression studies on a yellow cultivar demonstrating the absence of *DFR, FHT* and *ANS* expression due to the suppression of a bHLH transcription factor [8]. In the majority of white cultivars, in contrast, DFR alone was affected [3], which was also reflected by gene expression studies of 7 white and star-type cultivars [9].

Apart from anthocyanins and 6’-deoxychalcones, flavones and flavonols can be accumulated in dahlia flowers [10]. Despite being themselves almost colourless, they influence flower coloration as they interact with anthocyanins and thereby stabilize their structures, which are sensitive to oxidation and hydrolytic attack [11-14]. All cultivars tested showed a very high flavone synthase II (FNS II) activity, whereas flavonol synthase (FLS) activity was very low. Thus it was assumed, that flavones rather than flavonols are the predominant copigments present in dahlia flowers [3].

The base for the creation of black dahlia colour has not been investigated so far. We report, that the majority of black cultivars accumulate drastically decreased amounts of flavones, which is accompanied by low FNS II activities and suppressed *FNS II* expression. As flavone formation competes with anthocyanin biosynthesis for common intermediates, this results in the huge anthocyanin concentrations establishing the black cultivars.

## Results and discussion

### Pigment composition and enzyme activities in black cultivars

The petals of 14 black cultivars were analyzed for the presence of anthocyanins, flavonols and flavones in comparison to 1 white, 2 red and 2 yellow varieties. Acidic methanolic extracts from flower petals showed anthocyanin contents between 7 and 32 μmol/g FW (Table 1). Thus, the contents of black cultivars were above or at the end of the range reported previously for red cultivars [3]. In hydrolyzed extracts, both pelargonidin and cyanidin were present with varying ratios whereas no anthocyanidins were found in the yellow flowering cv. Rubens and cv. Alva’s Supreme or the white flowering cv. White Alva. The black cultivars showed diverse anthocyanidin composition with cyanidin proportions between 9% (cv. Black Barbara) and 80% (cv. Magic Night) (Table 1). Thus, black hues depend on the anthocyanin concentration rather than on the anthocyanin pattern. Black colouration of fruits and flowers is not frequent but has been studied in a few cases e.g. in cherry [11], lisanthus [12], viola [13], tulips [14], poppy [15], leaves of *Ophiopogon planiscapus*[16], and even in grains, such as black rice [17]. In all cases black tissues show large amounts of anthocyanins.

**Table 1:** Contents (μmol/g fresh weight) of anthocyanins, flavones and flavonols in different cultivars (nd: not determined)

**Cultivar**	**Colour**	**Anthocyanin content**	**Cy** %	**Flavone content**	**Flavonol content**
White Alva	white	-	-	30	8
Rubens	yellow	-	-	25	6
Alva’s Supreme	yellow	-	-	96	9
Feuerschein	bright red	2	15	68	12
Cheerio	red	1	30	8	1
Black Barbara	black	21	9	122	17
Arabian Night	black	19	59	5	23
Auroras Kiss	black	15	65	9	27
Black Jack	black	13	78	8	30
Chat Noir	black	8	72	3	14
Charles de Gaulle	black	10	57	3	6
Karma Choc	black	22	75	2	36
Kenora Macop B	black	10	65	nd	nd
Magic Night	black	18	80	22	34
Meteor	black	13	53	2	15
Nathal	black	8	78	6	16
Negerkopf	black	8	47	8	32
Stefanie Hertel	black	16	53	nd	nd
Tisa	black	33	58	1	31

Extracts of yellow, white and red cultivars contained large amounts of flavones (Table 1 and Additional file 1), which, in contrast, were present only in low amounts in the black cultivars. Flavonols were also present in white, yellow and red cultivars but compared to flavones only in low amounts thus confirming that flavones are the prevalent copigments in dahlia flowers [3]. In comparison to red cultivars, the flavonol content of black cultivars was increased (Table 1 and Additional file 1).

High activities of the enzymes of the anthocyanin pathway could be detected in enzyme preparations from petals of all black cultivars tested (Table 2). Yellow and white cultivars, in contrast, showed a low or a lack of DFR activity as expected. There was no general difference in the enzyme activities of red and black cultivars, but 3 of the black cultivars (cv. Chat Noir, Karma Choc and Tisa) showed a higher CHS activity than the red cultivars, which might increase the flux into the flavonoid pathway in these lines. However, 13 of 14 black cultivars were characterized by a low FNS II activity (Table 2). This was confirmed using microsomal preparations, in which membrane bound enzymes such as F3’H and FNS II are enriched. Using such preparations of petals from cv. Rubens, the FNS II assay was optimized with respect to pH and temperature optimum, temperature stability and linearity with time and protein. The results are summarized as standard enzyme assay for FNS II in petal preparations in the section material and methods. Incubation of microsomal preparations from the yellow cv. Rubens with eriodictyol in the presence of NADPH led to the formation of a single product which was identified as the flavone luteolin. Incubation with naringenin in the presence of NADPH led to the formation of the three products eriodictyol (flavanone), apigenin (flavone) and luteolin (flavone) as a result of the concerted activity of F3’H and FNS II. When microsomal preparations from black varieties were tested under the standard assay conditions, no or only a low product formation was observed when eriodictyol was used as a substrate (Additional File 2), while naringenin was converted to eriodictyol by F3’H.

**Table 2:** Enzyme activities in different cultivars

**Cultivar**	**Colour**	**CHS**	**FHT**	DFR nmol/s*kg fresh weight	**FNS II**	**F3**’**H**
White Alva	white	1.2 ± 0.1	1.8 ± 0.2	-	1.2 ± 0.13	1.8 ± 0.2
Rubens	yellow	4.1 ± 0.5	0.2 ± 0.0	-	2.1 ± 0.23	3.2 ± 0.4
Alva’s Supreme	yellow	1.7 ± 0.2	1.2 ± 0.1	-	2.0 ± 0.22	2.2 ± 0.3
Feuerschein	bright red	5.7 ± 0.6	3.0 ± 0.3	3.2 ± 0.4	1.1 ± 0.12	1.2 ± 0.1
Cheerio	red	5.0±0.6	1.5 ± 0.2	1.3 ± 0.2	1.4 ± 0.15	0.4 ± 0.1
Black Barbara	black	3.4 ± 0.4	3.1 ± 0.3	2.3 ± 0.3	1.3 ± 0.14	0.4 ± 0.1
Arabian Night	black	4.7 ± 0.5	2.8 ± 0.3	2.4 ± 0.3	0.3 ± 0.03	2.0 ± 0.2
Auroras Kiss	black	8.0 ± 0.9	3.0 ± 0.3	2.7 ± 0.3	0.1 ± 0.01	2.8 ± 0.3
Black Jack	black	4.9 ± 0.5	2.7 ± 0.3	2.1 ± 0.2	0.3 ± 0.03	2.8 ± 0.3
Chat Noir	black	9.8 ± 1.1	3.6 ± 0.4	1.5 ± 0.2	0.0 ± 0.00	3.6 ± 0.4
Charles de Gaulle	black	3.8 ± 0.4	2.2 ± 0.2	2.3 ± 0.3	0.3 ± 0.03	2.6 ± 0.3
Karma Choc	black	6.5 ± 0.7	2.7 ± 0.3	2.6 ± 0.3	0.2 ± 0.02	2.6 ± 0.3
Kenora Macop B	black	3.3 ± 0.4	2.2 ± 0.2	2.7 ± 0.3	0.1 ± 0.01	3.0 ± 0.3
Magic Night	black	3.4 ± 0.4	3.1 ± 0.3	2.6 ± 0.3	0.3 ± 0.04	3.0 ± 0.3
Meteor	black	3.0 ± 0.3	3.4 ± 0.4	2.7 ± 0.3	0.4 ± 0.04	2.4 ± 0.3
Nathal	black	2.0 ± 0.2	2.8 ± 0.3	2.2 ± 0.3	0.5 ± 0.05	3.5 ± 0.4
Negerkopf	black	4.3 ± 0.5	3.8 ± 0.4	3.0 ± 0.3	0.7 ± 0.07	2.6 ± 0.3
Stefanie Hertel	black	2.0 ± 0.2	2.4 ± 0.3	2.4 ± 0.3	0.5 ± 0.06	2.6 ± 0.3
Tisa	black	8.1 ± 0.9	3.5 ± 0.4	1.2 ± 0.1	0.1 ± 0.01	2.8 ± 0.3

The absence of FNS II activity correlates with the absence of flavones and increases the availability of flavanones such as naringenin and eriodictyol as precursors for anthocyanin formation. Thus, the low flavone formation favours the anthocyanin branch and thereby the accumulation of above-average amounts of anthocyanins found in black cultivars. Parallel to the decrease of flavones, flavonol contents slightly increased. The remaining flavones together with flavonols could serve as copigments for anthocyanin stabilization and intramolecular copigmentation could also be possible [18-21]. Actually, black colouration fades during petal aging and older petals appear dark red rather than black (Figure 1), an effect which is not observed in red cultivars. Compared to red cultivars, there is a drastic decrease in the molar ratio of copigments:anthocyanins in black cultivars, which might influence the colour stability [21].

### Cloning of cDNA clones and the corresponding genomic dahlia *FNS II*

To investigate the reasons for the lacking FNS II activity, we tested whether it was possible to isolate an *FNS II* cDNA clone from black cultivars. Primers (Table 3) were designed from conserved regions of *FNS II* sequences available in the NCBI database (Accession numbers BD270652-BD270670) and used for the isolation of a cDNA fragment from the petals from cv. Rubens. The full size cDNA clone was obtained by RACE techniques (GQ479808). Using specific primers, full size cDNA clones were obtained from the black cultivars Chat Noir (GQ489009, GQ489008) and Aurora’s Kiss (GQ489010). This indicated that the *FNS II* was expressed in black cultivars at least to some extent and that either pivotal mutations in the structural gene leading to loss of function or post-transcriptional or post-translational regulation or a too low expression level could be responsible for the lacking FNS II activity. 

**Table 3:** List of primers used

**Gene**	**Primer**		**Sequence 5’→3’**	**Fragment size (bp)**
	oligo(−dT) anchor primer		GACCACGCGTATCGATGTCGAC(T)16V	
*FNS II*	FNS1	F	CCCAACACCATGAATACACTCC	
	FNS4	R	TCACCACTGAGAGTTCTCTCATGG	
*FNS II*	FNSIIRub-ex	F	CCCAACACCATGAATACA C	
		R	GAAGCGAAGGTAAACACA C	
*FNS II*	Dah.FNSII.lang	F	CAACACCATGAATACACTCCTAGTACTCC	
		R	CTTAGACTGAAGTAAACATCGAATGTGGAGAG	
*FNS II*	qPCR	F	GCATCCAGAATCTCGGCCATT	
	qPCR	R	TCGTTGTCACTTCTCGCACTAG	
*Actin*	qPCR	F	GGAAAAGATTTGGCATCACACTTTC	103
	qPCR	R	AGCCTTAGGATTTAAAGGTGCCTCA	
*GAPDH*	qPCR	F	AGGCTGTTGGTAAGGTGTTGC	141
	qPCR	R	GCAGCCTTGATCTGCTCATAAG	
*CHS1*	qPCR	F	GCGCGTATATGGCACCTTCG	198
	qPCR	R	GGCCGAGGAGCTTGGTGAGT	
*CHS2*	qPCR	F	ACGTTTCGTGGACCGACGGAT	185
	qPCR	R	CAGCCCCACCTCCCTCAAG	
*CHI*	qPCR	F	ACTTCCCTCCCGGCTCCTCT	182
	qPCR	R	TTGTTTGGCTGCTGGGGAAACAC	
*FHT*	qPCR	F	CCACAGCCCGACCTGACACT	202
	qPCR	R	CGTTCTTGAACCGCCCATTG	
*DFR*	qPCR	F	GCCAAAGCCAAGACGGTCAA	192
	qPCR	R	CGTTGCCTTCCATGCTGCTT	
*ANS*	qPCR	F	GCTGCAGGCGAGGGTTTCTT	119
	qPCR	R	GTCGACGGCCAAATGGTGAG	
*F3’H*	qPCR	F	AACTGCCTTGCTATTGTACGTACTG	147
	qPCR	R	TTTTCCGCCAGGGCTGCC	
MaT	qPCR	F	TTGGAATATGCCGACCGATG	112
	qPCR	R	CCTTTCCCCACCCAAAATCA	
*Delila*	qPCR	F	ATCTCACAAACGCCGAGTGGTATT	158
	qPCR	R	TGCTAGAAGAGAGCGGCAGAAGA	
*IVS*	qPCR	F	CGTCGTCGAGAGAAGCTAAACGA	151
	qPCR	R	GACATCGTGCTTCCAAATCCTGAA	
*MYB1*	qPCR	F	TTGAGGAAAGGTTCATGGTCAGC	145
	qPCR	R	AATTTGTCCATCGTAGCCTGCAAC	
*MYB2*	qPCR	F	ACCCTCCTCCAACCACTCATCAT	160
	qPCR	R	TGAGGTGGGCTCATTCTCAACTC	
*R3MYB*	qPCR	F	ATGGCCGTGAGAAGGAAATTGATG	153
	qPCR	R	CAAGGAGGTCCAACATGACAAC	
*WDR1*	qPCR	F	CGGTTGCGGAACTAGAGAGAC	155
	qPCR	R	TGACATCGGGTCAATCCCATTAGG	
*WDR2*	qPCR	F	CAGAGGCATCAGTCGAGTGTGA	149
	qPCR	R	GCCAATATCGGATCCAATCCACCT	

Abbrev.: F: forward, R: reverse.

**Figure 2:**
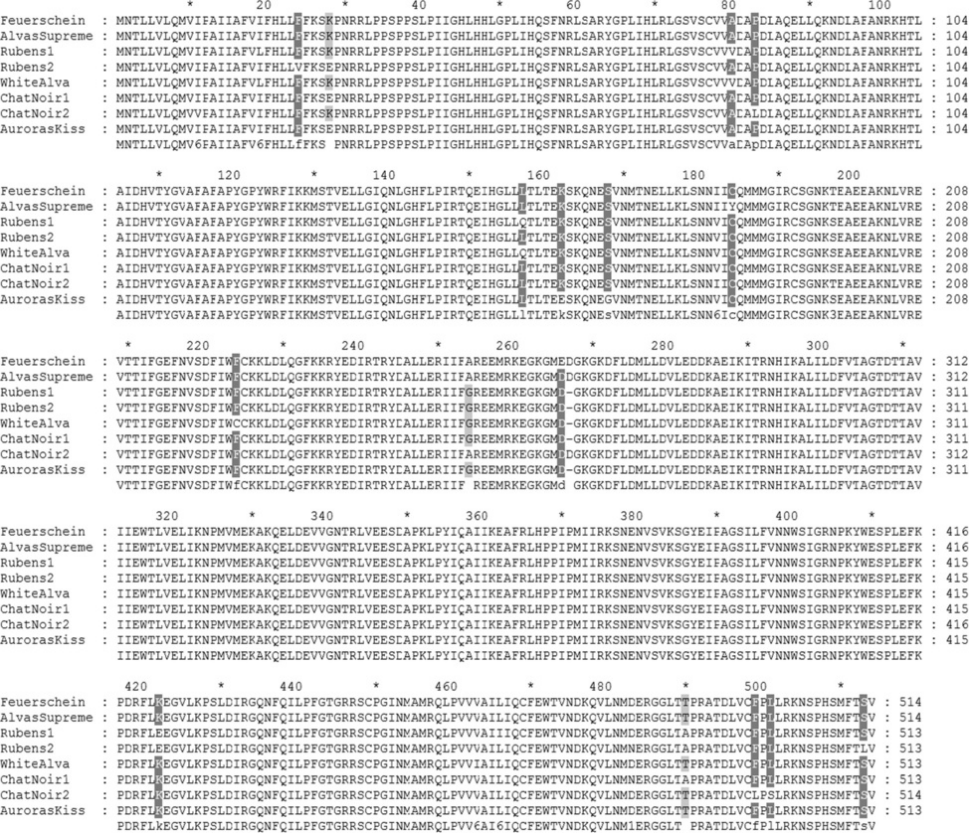
**Multiple amino acid sequence alignment of *FNS II*.** Alignment was performed with GeneDoc [38] and provides a comparison of the cDNA clones obtained from the red cultivar Feuerschein (GQ479806), cv. White Alva (GQ489011), yellow cultivars Alva’s Supreme (GQ479807), cv. Rubens (GQ479808, GQ479809) and the black cultivars Chat Noir (GQ489009, GQ489008) and Aurora’s Kiss (GQ489010). Low consensus sites are shaded in grey.

The sequences were highly conserved and showed 97-99% similarity at the amino acid level (Figure 2). From petals of cv. Rubens and cv. Chat Noir two alleles were found showing 98% similarity at the amino acid level, respectively. Further cDNA clones were obtained from yellow cultivars Rubens (GQ479809) and Alva’s Supreme (GQ479807), the red cultivar Feuerschein (GQ479806), the white cultivar White Alva (GQ489011). From sequence comparison, no discrimination according to flower colour could be found.

**Figure 3:**
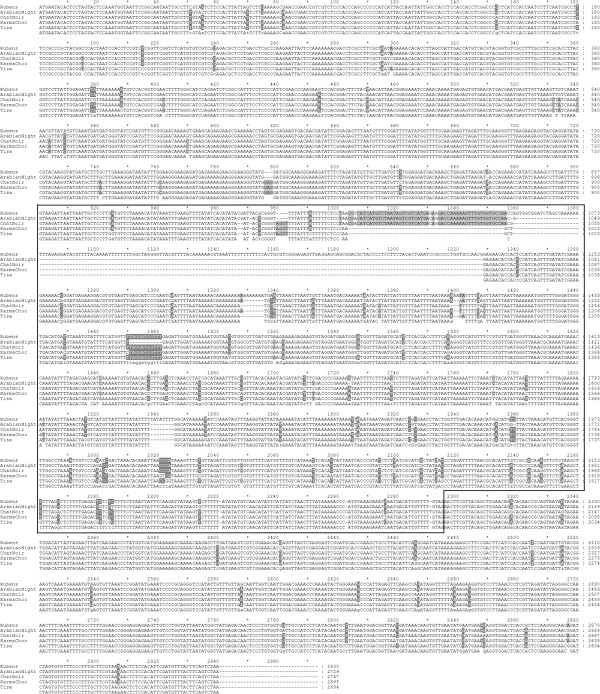
**Multiple alignments of genomic *FNS II *nucleotide sequences of *Dahlia variabilis *hort.** Alignment was performed with GeneDoc [38] and provides a comparison of *FNS II* of the yellow cv. Rubens (Accession No JQ731761) and of the black cultivars Arabian Night (Accession No JQ731764), Chat Noir (Accession No JQ731765), Karma Choc (Accession No JQ731763), and Tisa (Accession No JQ731762). Low consensus sites are shown in black letters and shaded in grey, others in white letters and shaded in grey. The intron containing region is boxed.

In addition, the corresponding genomic clones of *FNS II* of the yellow cv. Rubens (Accession No JQ731761) and of the black cultivars Arabian Night (Accession No JQ731764), Chat Noir (Accession No JQ731765), Karma Choc (Accession No JQ731763), and Tisa (Accession No JQ731762) were isolated. The genomic clones had one intron at the same position (889 in Accession No JQ731761, Rubens). However the intron length differed considerably (Figure 3). Thus, the clones had the size of 2939 bp (Accession No JQ731761), 2704 bp (Accession No JQ731762), 2702 (Accession No JQ731763), 2736 bp (Accession No JQ731764), and 2757 bp (Accession No JQ731765).

### Functional activity and characterization of recombinant enzymes

To investigate whether the small differences in the *FNS II* sequence could be relevant for the establishment of black phenotypes, all cDNA clones were heterologously expressed in *S. cerevisiae*. As assumed, the obtained recombinant proteins were functionally active and catalyzed the NAPDH-dependent formation of apigenin and luteolin from naringenin and eriodictyol, respectively, thus confirming that the differences in the sequence are not pivotal. Recombinant FNS II of the yellow cv. Rubens (GQ479808) and the black cv. Chat Noir (GQ489009) were characterized in more detail and largely showed the same characteristics as expected from the high sequence similarity. The optimal pH was 6.50 for the conversion of naringenin and pH 7.50 for eriodictyol as substrates. The enzyme was stable until 25°C and the temperature optimum was 25-30°C. The reaction was linear with time until 25 min and with the protein content up to 31 μg (GQ479808) or 15 μg (GQ489009) in the assay. Kinetic studies showed apparent *K*_*m*_ and *V*_*max*_ values of 3.86 μM and 22.7 μmol/s*kg for naringenin as substrate and 4.2 μM and 22.3 μmol/s*kg for eriodictyol as substrate, resulting in a ratio of *V*_*max*_/*K*_*m*_ of 5.9 for naringenin and 5.3 for eriodictyol, respectively. Thus, the FNS II shows comparably high specificity for both substrates.

### Gene expression studies

**Figure 4:**
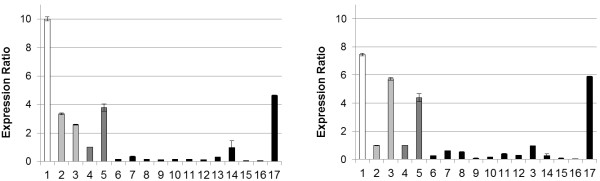
**Relative expression of *FNS II *in the petals of differently coloured dahlia cultivars.***FNS II* expression was measured in comparison to the housekeeping genes *Actin* (left) *GAPDH* (right) in the white cv. White Alva (1), yellow cvs. Rubens (2) Alva’s Supreme (3), red cvs. Feuerschein (4), Cheerio (5) and black cvs. Arabian Night (6), Auroras Kiss (7), Black Jack (8), Charles de Gaulle (9), Chat Noir (10), Karma Choc (11), Magic Night (12), Meteor (13), Mythos (14), Negerkopf (15), Tisa (16), Black Barbara (17). The relative gene expression was calculated in comparison to the red cv. Feuerschein.

The expression of structural and regulatory genes from the flavonoid pathway was measured by quantitative Real-time PCR in comparison to two housekeeping genes, *actin* and *glyceraldehyde 3-phosphate dehydrogenase* (*GAPDH)*. The expected size of the amplicons was confirmed by gel electrophoresis and the specificity by melt curve analysis. *FNS II* was expressed in all cultivars. However, black cultivars showed a very low *FNS II* expression in comparison to the other cultivars (Figure 4), with the exception of cv. Black Barbara, which showed second highest *FNS II* expression in accordance with the relatively high FNS II activities measured (Table 2). White and yellow cultivars showed higher *FNS II* expression than the red cv. Feuerschein and highest expression was observed in the cv. White Alva (Figure 4). This correlates well with the observed low FNS II activity in the majority of black cultivars. As *D. variabilis* is octoploid (*2n*=*8x*=*64*) [22], it is expected that more than one *FNS II* copy is present as recently shown for *D. variabilis CHS*[9]. The decline of FNS II activity in the majority of the black cultivars, which is unusual for dahlia [3], indicates the presence of an effective regulatory mechanism, which could also include post-transcriptional or post-translational processes.

An impact of the flavone pathway on anthocyanin accumulation and flower colour was shown in several studies. *Gerbera* lines lacking *FNSII* expression and FNSII activity did not accumulate flavones, while anthocyanin contents and intensity of the flower colour increased noticeably [23]. However, the anthocyanin concentrations obtained were not high enough to result in black flowers. Vice versa, the transformation of *FNSII* into violets led to the accumulation of flavones at the expense of anthocyanins, which showed paler flowers than the wild type. Daphne mutants of *Antirrhinum majus*, which lack flavones, develop dull reddish-brown flowers in contrast to red wild type flowers [24].

**Figure 5:**
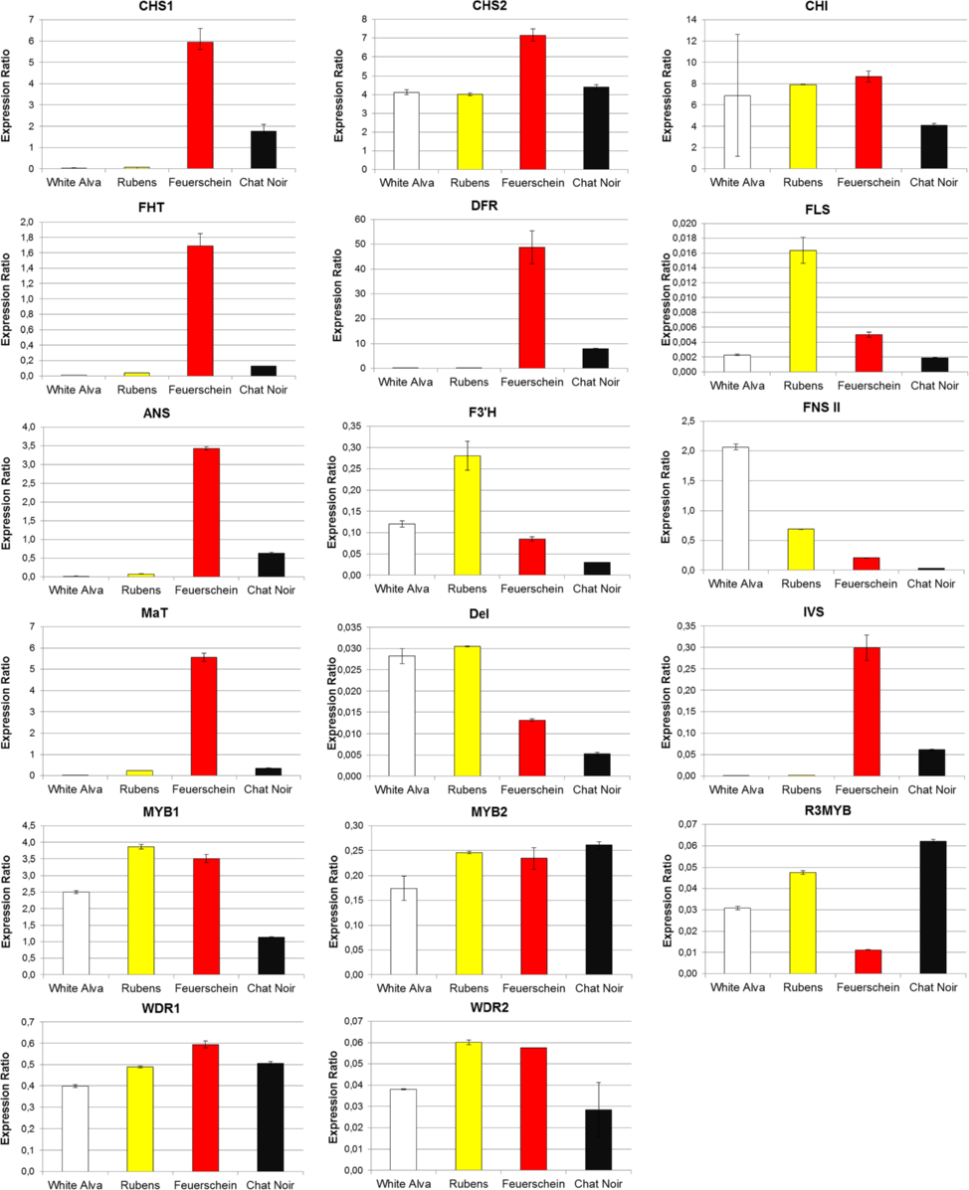
**Expression of structural genes and transcription factors in *D. variabilis *cultivars of four different colour types.** Expression was measured in comparison to the housekeeping genes *Actin* in the white cv. White Alva, yellow cvs. Rubens, red cv. Feuerschein and black cv. Chat Noir.

To investigate the possible involvement of transcription factors from *D. variabilis* on the *FNS II* suppression, we determined the expression of seven recently identified putative regulatory genes *MYB1, MYB2, R3MYB, WDR1, WDR2* and the bHLH transcription factors *DEL* and *IVS*[8,9,25]. Interaction of comparable proteins resulted in the formation of a regulatory complex that controls cellular identity of epidermal cells including anthocyanin formation [26-28]. All 14 black cultivars showed a similar expression pattern (Additional Files 3 and 4). For a better overview and comparison between the colour types the relative expression pattern in the white cv. White Alva, the yellow cv. Rubens, the red cv. Feuerschein and the black cv. Chat Noir is shown in Figure 5. The expression pattern of the transcription factors followed the same trend in the red and black cultivars and no general difference could be found explaining the black phenotype, although some black cultivars occasionally showed a lower expression e.g. cv. Chat Noir for *MYB1* or cv. Mythos for *MYB2*. The only difference related to colour was the suppressed *IVS* expression in the yellow and white cultivars as previously reported [8]. Thus, the suppression of *FNS II* expression must be influenced by other so far unknown factors. Changes in the promoter regions are unlikely to play a particular role because several alleles must be affected at the same time.

Ohno et al. [9] have shown the presence of two different chalcone synthases in dahlia with several alleles of each type. *CHS 1* seems to be specifically involved into anthocyanin formation because it shows a similar expression pattern as *FHT*, *DFR* and *ANS* as a result of *IVS* expression and is suppressed in white and yellow cultivars. *CHS 2* in contrast was expressed independently from the flower colour [9]. This was also confirmed in our study. *CHS 1* was expressed in the red and all black cultivars but not in the white and yellow ones, where *CHS 2* was expressed in all cultivars (Figure 5, Additional Files 3 and 4). It may be speculated that the two CHS types were introduced into the *D. variabilis* genome by the two different wild species which are the parent plants of the garden dahlia [29,30]. So far the presence of isoenzymes in *D. variabilis* was only observed for CHS. However, our data suggest that also more than one *FHT* gene exists, because the FHT enzyme activity measured in the yellow cv. Rubens and the white cv. White Alva was higher than expected from the *FHT* suppression observed in the white and yellow cultivars (Figure 5, Table 2). In addition, other dioxygenases from the flavonoid pathway such as FLS, which has been shown to accept a broad range of substrates [31], could contribute to the observed FHT activity in these lines.

The relative expression of the structural genes from the flavonoid pathway was largely comparable and sometimes even lower than those of the red cultivars. This underpins that the increased anthocyanin formation in the majority of black cultivars is not based on an induced anthocyanin pathway, but exclusively the result of the promoted flux of flavanone intermediates into anthocyanins due to the reduced flavone formation. Only in the case of cv. Black Barbara, there is huge anthocyanin formation despite unhampered flavone formation. On the other hand, this cultivar shows highest total expression of *CHS* genes (*CHS1* and *2*) and is also very high for all the other genes. This could be an explanation for the high anthocyanin formation, but we cannot exclude that there are further, so far unknown relevant factors. Expression of *malonyl-coenzyme A:anthocyanidin 3-O-glucoside-6”-O-malonyltrans-ferase* (*MaT*) varied largely within the black cultivars and no correlation with flower colour was found (Figure 5, additional files 3 and 4). Thus, increased anthocyanin stability by malonylation of anthocyanins does not seem to be play a role for the establishment of the black phenotypes. Interestingly, *malonyltransferase* showed a similar suppression as *CHS 1, FHT*, *DFR* and *ANS* in yellow and white varieties indicating that the gene could be coregulated with the other genes distinctly involved in the formation of red pigmentation.

## Conclusions

**Figure 6:**
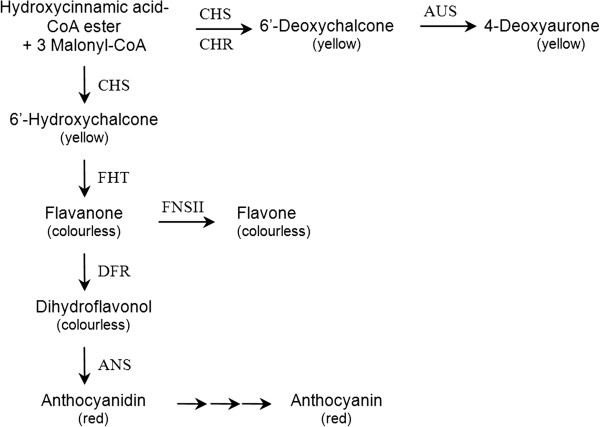
**Overview of the flavonoid pathway in *D. variabilis*.** In black cultivars, branches leading to yellow 6’-deoxychalcones and 4-deoxyaurones and colourless flavones are blocked and low amounts of flavonols are formed. Flux into anthocyanin formation is considerably enhanced. In white and yellow cultivars the pathway to anthocyanins is commonly blocked at the level of DFR.

Continuous dahlia breeding worldwide has led to the availability of a countless number of cultivars, many of them showing red hues. However, black hues of dahlia flowers seem to occur only occasionally. We have shown for the first time that the distinct impression of black colour in dahlia is based on the accumulation of huge amounts of anthocyanins. In the majority of the black cultivars, this seems to be a result of increased channelling of flavanones towards anthocyanins at the expense of flavone formation (Figure 6). This seems to be based on the low FNS II activity and *FNS II* expression which is not influenced by any transcription factor of the flavonoid pathway known so far. Whereas the flavonoid pathway offers a large spectrum of possibilities for the establishment of white, yellow, orange, pink and red tones, 11 of 12 cultivars showed an identical biochemical and molecular background establishing the phenotype. The cv. Black Barabara’, however, showed a generally high expression of genes from the anthocyanin pathway, which could provide an explanation for the high amounts of anthocyanins found despite high flavone formation. A higher substrate supply due to changes in the phenylpropanoid pathway could also result in enhanced anthocyanin formation. Further investigations will be required to understand the establishment of the black colour in the special case of cv. Black Barbara. The molecular explanation for the specific suppression of flavone formation in the majority of black dahlia will be of particular interest. As dahlia is an octoploid plant and the presence of several alleles is expected, the simultaneous suppression of all FNS II isoenzymes indicates an effective mechanism, which could be used for engineering plants with tailor-made flavone contents.

## Methods

### Plant material

*Dahlia variabilis* hort. cv. Arabian Night, cv. Karma Choc and cv. Tisa were obtained from Kubelka (Spitz a.d. Donau, Austria), cv. Auroras Kiss, cv. Black Jack, cv. Magic Night and cv. Negerkopf from Haslhofer (Windischgarsten, Austria), cv. Black Barbara from Aviflora (Aalsmeer, Netherlands), cv. Chat Noir, cv. Charles de Gaulle, cv. Kenora Macop B, cv. Rubens, cv. Alva’s Supreme, cv. Feuerschein, cv. Cheerio, cv. White Alva from Dahlien-Kultur Wirth (Vienna, Austria), cv. Meteor, cv. Stefanie Hertel from Paul Panzer (Bad Köstritz, Germany) and cv. Nathal from Dahlien Schwieter (Legden, Germany). According to previous studies [3], opening buds with a maximal petal length of 2.5 cm were used for the determination of gene expression, enzyme activity and analysis of the presence of flavonoid classes. Determination of anthocyanin contents was additionally performed with extracts from fully developed flowers. Due to the specific shape of inflorescences from Asteraceae species, all samples are a mixture of petals of all size and age present in the respective developmental flower stage. All samples were taken from at least 10 plants. All investigations were done on at least three biological replicates. The plant material was shock frozen in liquid nitrogen and stored at −80°C.

### Enzyme preparations

Petals were ground in a mortar with 0.25 g Polyclar AT, 0.25 g quartz sand and 3 ml 0.1 M Tris/HCl (pH 7.25, containing 0.4% sodium ascorbate) and then centrifuged for 3 min at 10,000 x g. FNS II and F3’H assays were performed using the supernatant or microsomal preparations obtained according to [32]. For the determination of other enzymes, preparations were subjected to a gel chromatography step (Sephadex G25,medium, GE Healthcare).

### Chemicals

^14^C]malonyl-CoA was purchased from Amersham International (Freiburg, Germany), *p*-coumaroyl-CoA from TransMIT (Marburg, Germany), apigenin, luteolin, butein, kaempferol, quercetin, and 3’,5,7-trihyroxy-3,4’-dimethoxyflavone from Extrasynthese (Genay, France). ^14^C]Naringenin, and ^14^C]eriodictyol were synthesized as described [32].

### Enzyme assays

Enzyme assays were performed according to [3]. Quantification of protein was done with a modified method of Lowry [33]. The standard assay for FNS II contained in a final volume of 100 μl 0.046 nmol ^14^C]flavanone, 20 μl microsomal preparation, 5 μl 1 mM NADPH and 75 μl 0.1 M KH_2_PO_4_ containing 0.4% Na-ascorbate (pH 6.75). The reaction was stopped with 70 μl EtOAc and 10 μl acetic acid. The organic phase was transferred to thin-layer cellulose plates (Merck, Darmstadt, Germany) and developed in CAW (chloroform/acetic acid/water: 10/9/1). The evaluation was carried out on a Berthold LB 2842 TLC linear analyzer (Wildbad, Germany) by integration of the peak areas.

### Pigment analysis

For the determination of the anthocyanin content, 0.5 g of shock-frozen petals were pulverized and mixed with 2.5 ml 2 M methanolic hydrochloric acid. After shaking for 120 minutes in an overhead rotator, the suspension was centrifuged for 10 minutes at 19200 x g. 10–140 μl of the supernatant was adjusted with 2 M methanolic hydrochloric acid to a final volume of 1000 μl. The absorption at 520 nm was determined on a DU-65 spectrophotometer (Beckman Instruments). The anthocyanin content was calculated as pelargonidin equivalent using a calibration curve obtained with commercially available pelargonidin chloride (Roth, Germany).

For acidic hydrolysis of anthocyanins, 20 μl methanolic hydrochloric acid extract were mixed with 180 μl of 4 N HCl and incubated for 60 minutes at 90°C. After cool-ing for 10 minutes the mixture was centrifuged for 10 minutes at 10,000 x g. The supernatant was adjusted to 200 μl with 4 N HCl and aliquots were used for HPLC analysis. For enzymatic hydrolysis of flavones and flavonols, 20 μl of the methanolic extract was incubated for 10 min at 40°C with 10 U naringinase (Sigma-Aldrich, Austria) dissolved in 80 μl 0.1 M McIlvainebuffer, pH 4.0. The reaction was stopped by addition of 40 μl methanol and subjected to HPLC analysis. HPLC analysis was performed according to [34] using a Perkin Elmer Series 200 HPLC system equipped with a Perkin Elmer Series 200 diode array detector and Total Chrom Navigator, version 6.3.1 (Perkin Elmer Inc). The column was a BDS Hypersil C18 HPLC column, 5 μm, 250 x 4.6 mm (Thermo Scientific). For quantification 3’,5,7-trihydroxy 3,4’- dimethoxyflavone was used as internal standard.

### Cloning the corresponding FNS II cDNA clones and genomic *FNS II*

mRNA was isolated from *dahlia* petals, using the μMACS mRNA Isolation Kit (Miltenyi Biotec, Bergisch-Gladbach, Germany). cDNA was prepared using the RevertAid H Minus MuLV reverse transcriptase (Fermentas Life Science, St. Leon-Rot, Germany) with an oligo(−dT) anchor primer (Table 3). Primers for the isolation of *FNS II* from dahlia were designed using *FNS II* sequences, which were available in the NCBI GenBank (BD270652-BD270670, AF156976, AF188612). Primers outside the coding sequence were chosen (FNS1 and FNS4, Table 3). The obtained cDNA-fragments were isolated, ligated into the vector pCR®2.1-TOPO (Invitrogen, Paisley, UK), transformed in *E.coli* (TOP10, Invitrogen Paisley, UK) and sequenced by a commercial supplier (StarSEQ, Mainz, Germany). The obtained sequence information was used for the design of specific primers (FNSIIRub-ex, Table 3). Proofreading amplification of the complete open reading frame was carried out using the Expand High Fidelity PCR System (Roche, Mannheim, Germany). For heterologous expression in yeast, proofreading cDNA amplicons were ligated into the pYES2.1/V5-His-TOPO® vector (Invitrogen, Paisley, UK). Sense constructs were isolated and confirmed by sequencing. Transformation of the plasmid into the *S. cereviasiae* strain INV*Sc*1 followed. Heterologous expression and preparation of the recombinant protein was carried out according to [35].

Genomic DNA was prepared using the DNeasy Plant Mini Kit (Qiagen, Hilden, Germany) according to the supplier’s instruction. Specific primers (Dah.FNSII.lang, Table 3) for the amplification of the genomic *FNS II* were designed from the cDNA sequence information.

### Gene expression studies

Expression of *FNS II* was quantified by qPCR using a StepOnePlus system and SYBR® Green PCR Master Mix (Applied Biosystems, Darmstadt, Germany) according to the supplier’s instruction. The analysis was carried out in triples, and the data of *FNS II* was normalized against two control genes, *actin* and *glyceraldehyd 3-phosphate dehydrogenase* (*GAPDH*). Primers were derived from [36] for *Actin, CHI, CHS1, CHS2, FHT, ANS, FLS, Delila, Myb1, Myb2, R3Myb, WDR1, WDR2,* and *IVS* and from [37] for *F3’H*. Primers for *DFR* were based on the sequence published in the NCBI database (FJ216425), which was previously derived using degenerated primers for conserved DFR sequences. Primers for *MaT* were based on the sequence published in the NCBI database (AF489108). Primers for *GAPDH* were designed based on the conserved regions in mRNA sequences of *GAPDH* of different Asteraceae species published in the NCBI-GenBank (EU708566, AF162198, GU475487). The sequences of the primers are shown in Table 3.

## Abbreviations

ANS: Anthocyanidin synthase; bHLH: Basic helix-loop-helix; CHS: Chalcone synthase; CHI: Chalcone isomerase; Cy: Cyanidin; DFR: Dihydroflavonol 4-reductase; EtOAc: Ethyl acetate; F: Forward; F3’H: Flavonoid 3’-hydroxylase; FHT: Flavanone 3-hydroxylase; FLS: Flavonol synthase; FNS II: Flavone synthase II; GAPDH: Glyceraldehyde 3-phosphate dehydrogenase; HPLC: High-performance liquid chromatography; MaT: Malonyl-coenzyme A:anthocyanidin 3-O-glucoside-6”-O-malonyltrans-ferase; NAPDH: Nicotinamide adenine dinucleotide phosphate; R: Reverse; RACE: Rapid amplification of cDNA ends; TLC: Thin layer chromatography; WDR: WD40 repeats.

## Competing interests

The authors declare that they have no competing interests.

## Authors’ contributions

GM screened the dahlia cultivars for the presence of flavonoid enzymes and showed that black dahlia does not possess FNS II activity, JT isolated the genomic clones, performed the quantitative Real-time PCR studies and contributed to the preparation of the manuscript. KSL performed the isolation and characterization of the cDNA clones. SM showed the functional activity, characterized the recombinant enzymes, analyzed the presence of flavonoids in the petals and supervised the work of RA who characterized the FNS II activity in plant preparations and contributed to all other parts of the work. KST participated in the design of the study and the manuscript drafting. HH conceived the study, participated in all parts of work and drafted the manuscript. All authors read and approved the final manuscript.

## Supplementary Material

Additional file 1 **Proportional amounts of flavones, flavonols and anthocyanins in the white cv.** White Alva (1), yellow cvs. Rubens (2) Alva’s Supreme (3), red cvs. Feuerschein (4), Cheerio (5),and black cvs. Arabian Night (6), Auroras Kiss (7), Black Jack (8), Chat Noir (9), Charles de Gaulle (10), Karma Choc (11), Magic Night (12), Meteor (13), Natal (14), Negerkopf (15), Tisa (16), Black Barbara (17). For absolute values refer to Table 1. Click here for file

Additional file 2 **Flavone synthase II assay in three black cultivars.** Radiochromatogram of TLC on cellulose with solvent system CAW (chloroform/acetic acid/water: 10/9/1) from incubation of [^14^C]eriodictyol in the presence of microsomal preparations from petals of cv. Black Barabara (left), cv. Chat noir (centre) and cv. Stefanie Hertel (right). Click here for file

Additional file 3 **Relative expression of structural genes and transcription factors.** Expression was determined in comparison to the housekeeping gene *actin* in the white cv. White Alva (1), yellow cvs. Rubens (2) Alva’s Supreme (3), red cvs. Feuerschein (4), Cheerio (5), and black cvs. Arabian Night (6), Auroras Kiss (7), Black Jack (8), Charles de Gaulle (9), Chat Noir (10), Karma Choc (11), Magic Night (12), Meteor (13), Mythos (14), Negerkopf (15), Tisa (16), Black Barbara (17). Click here for file

Additional file 4 **Relative expression of structural genes and transcription factors.** Expression was determined in comparison to the housekeeping gene *glyceraldehyde 3-phosphate dehydrogenase* (*GAPDH*) in the white cv. White Alva (1), yellow cvs. Rubens (2) Alva’s Supreme (3), red cvs. Feuerschein (4), Cheerio (5), and black cvs. Arabian Night (6), Auroras Kiss (7), Black Jack (8), Charles de Gaulle (9), Chat Noir (10), Karma Choc (11), Magic Night (12), Meteor (13), Mythos (14), Negerkopf (15), Tisa (16), Black Barbara (17). Click here for file
