# Supplementary figures and images for: 'Le Rouge et le Noir': A decline in flavone formation correlates with the rare color of black dahlia (Dahlia variabilis hort.) flowers
Source: BMC Plant Biol. 2012 Nov 23;12:225. doi: 10.1186/1471-2229-12-225 (PMC3557166; doi:10.1186/1471-2229-12-225)

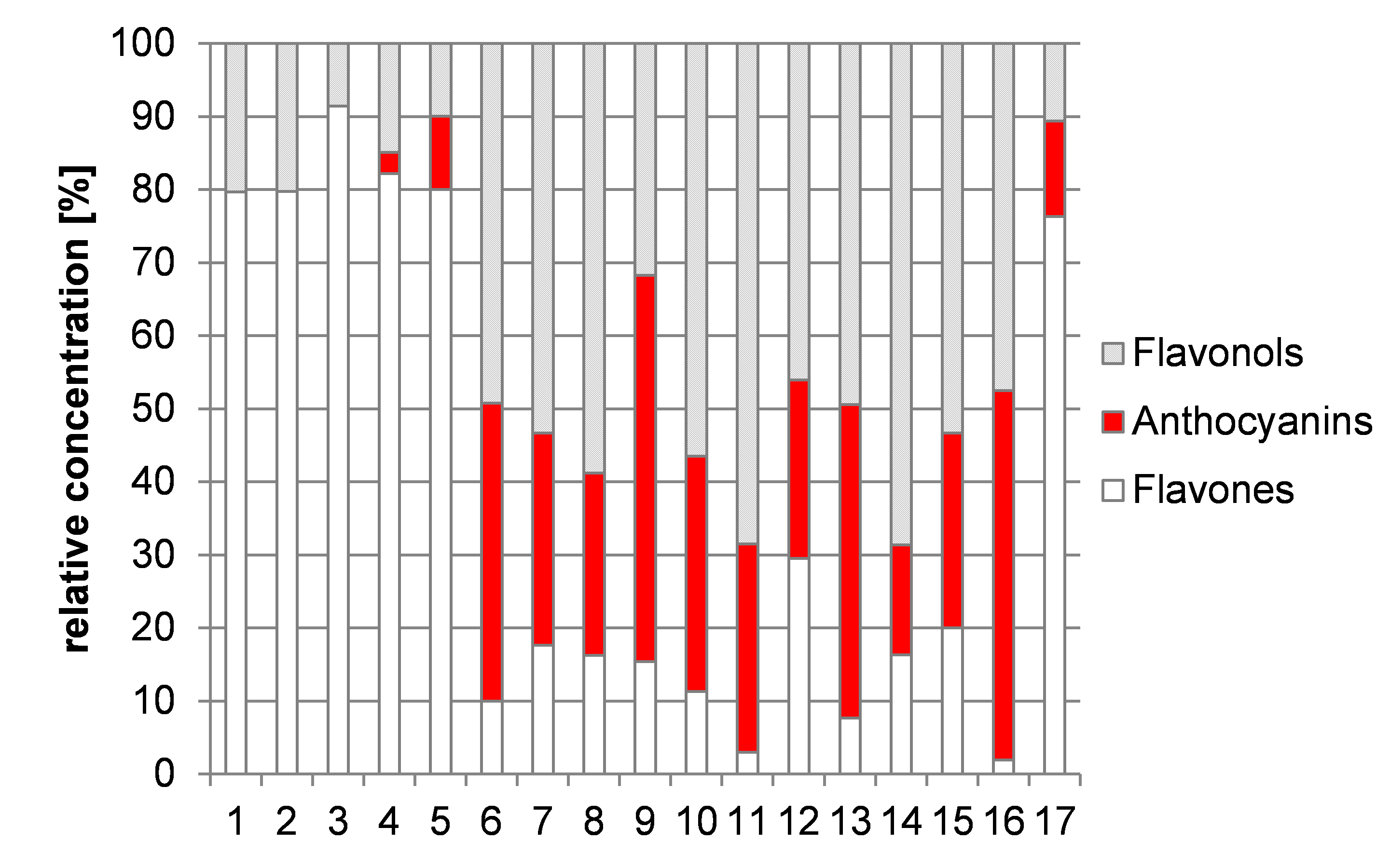

Supplement: Additional file 1 — Proportional amounts of flavones, flavonols and anthocyanins in the white cv. White Alva (1), yellow cvs. Rubens (2) Alva’s Supreme (3), red cvs. Feuerschein (4), Cheerio (5),and black cvs. Arabian Night (6), Auroras Kiss (7), Black Jack (8), Chat Noir (9), Charles de Gaulle (10), Karma Choc (11), Magic Night (12), Meteor (13), Natal (14), Negerkopf (15), Tisa (16), Black Barbara (17). For absolute values refer to Table 1. [file 1471-2229-12-225-S1.tiff]

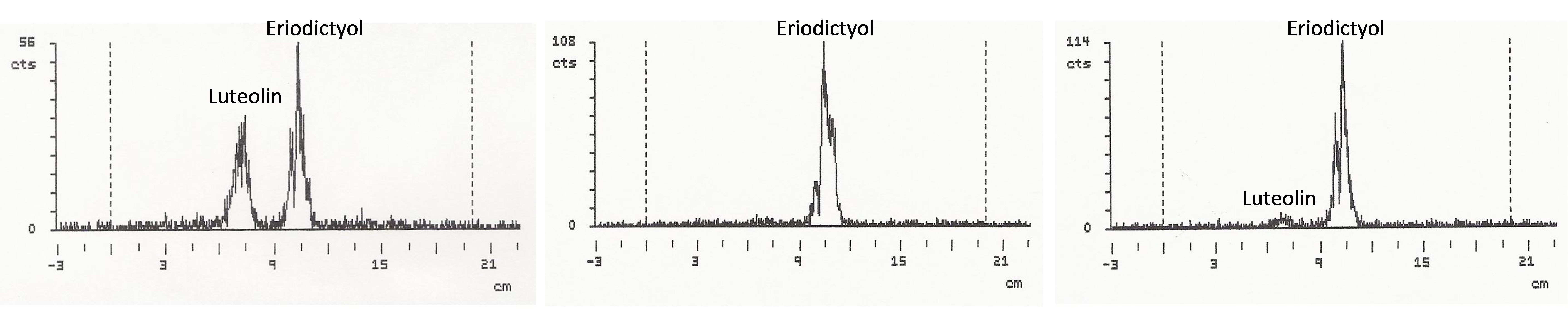

Supplement: Additional file 2 — Flavone synthase II assay in three black cultivars. Radiochromatogram of TLC on cellulose with solvent system CAW (chloroform/acetic acid/water: 10/9/1) from incubation of [14C]eriodictyol in the presence of microsomal preparations from petals of cv. Black Barabara (left), cv. Chat noir (centre) and cv. Stefanie Hertel (right). [file 1471-2229-12-225-S2.tiff]

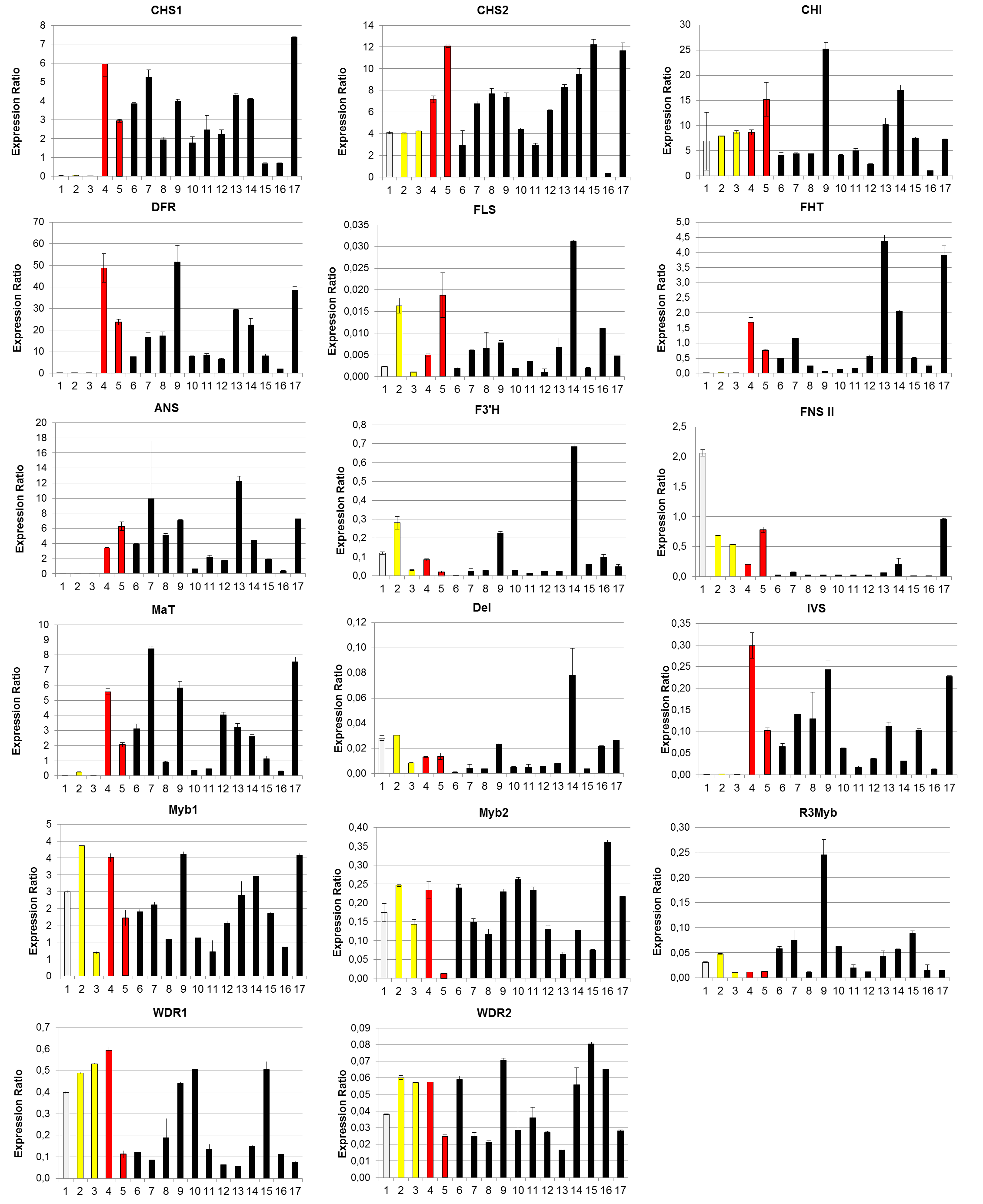

Supplement: Additional file 3 — Relative expression of structural genes and transcription factors. Expression was determined in comparison to the housekeeping gene actin in the white cv. White Alva (1), yellow cvs. Rubens (2) Alva’s Supreme (3), red cvs. Feuerschein (4), Cheerio (5), and black cvs. Arabian Night (6), Auroras Kiss (7), Black Jack (8), Charles de Gaulle (9), Chat Noir (10), Karma Choc (11), Magic Night (12), Meteor (13), Mythos (14), Negerkopf (15), Tisa (16), Black Barbara (17). [file 1471-2229-12-225-S3.tiff]

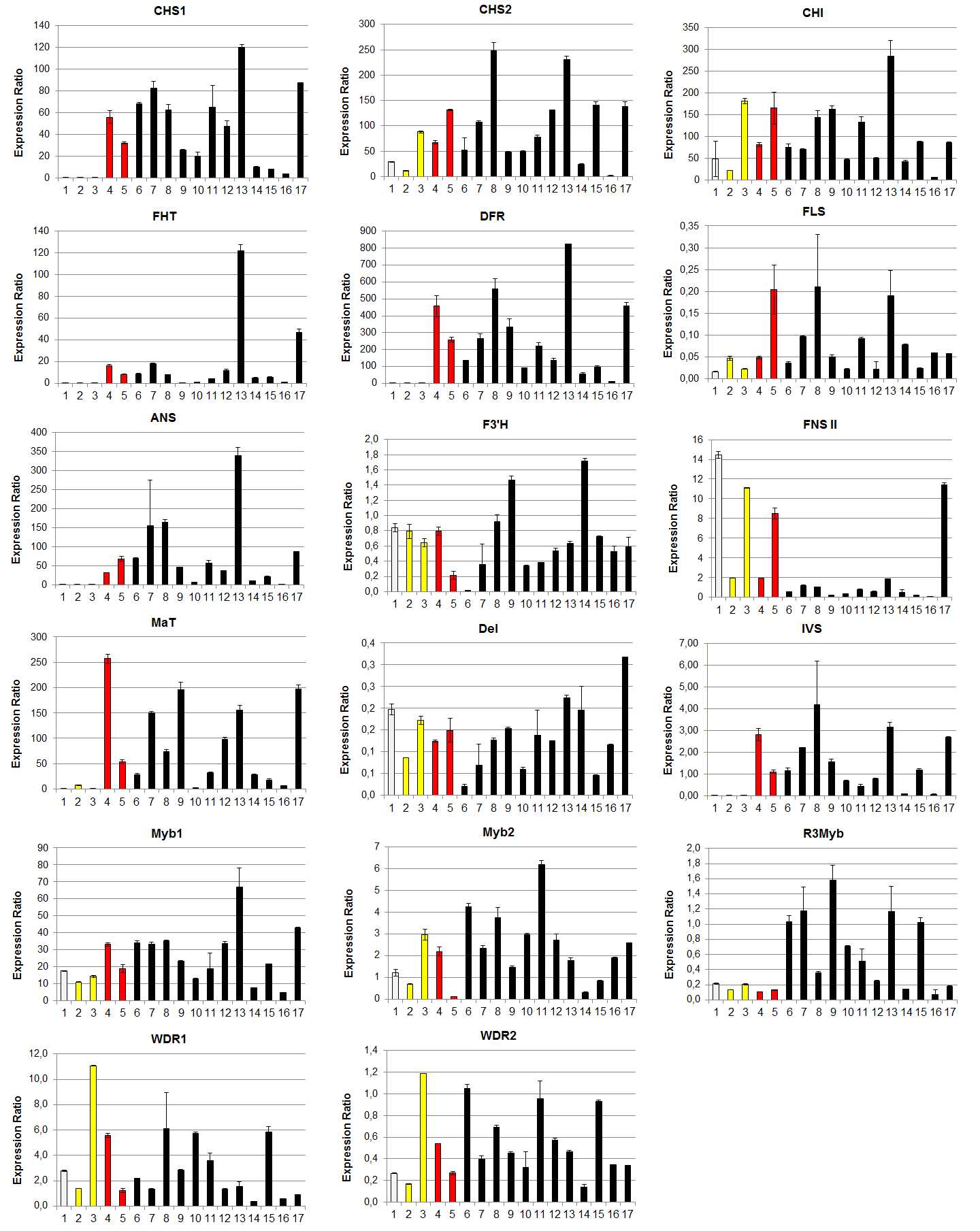

Supplement: Additional file 4 — Relative expression of structural genes and transcription factors. Expression was determined in comparison to the housekeeping gene glyceraldehyde 3-phosphate dehydrogenase (GAPDH) in the white cv. White Alva (1), yellow cvs. Rubens (2) Alva’s Supreme (3), red cvs. Feuerschein (4), Cheerio (5), and black cvs. Arabian Night (6), Auroras Kiss (7), Black Jack (8), Charles de Gaulle (9), Chat Noir (10), Karma Choc (11), Magic Night (12), Meteor (13), Mythos (14), Negerkopf (15), Tisa (16), Black Barbara (17). [file 1471-2229-12-225-S4.tiff]
